# Helper-Like Type-1 Innate Lymphoid Cells in Inflammatory Bowel Disease

**DOI:** 10.3389/fimmu.2022.903688

**Published:** 2022-06-23

**Authors:** Diana Coman, Isabelle Coales, Luke B. Roberts, Joana F. Neves

**Affiliations:** ^1^Centre for Host Microbiome Interactions, King’s College London, London, United Kingdom; ^2^School of Immunology and Microbial Sciences, King’s College London, London, United Kingdom

**Keywords:** innate lymphocyte cells, intestine, inflammatory bowel disease, inflammation, natural killer cell, type 1 innate lymphoid cells

## Abstract

Inflammatory bowel disease (IBD) is an idiopathic condition characterized by chronic relapsing inflammation in the intestine. While the precise etiology of IBD remains unknown, genetics, the gut microbiome, environmental factors, and the immune system have all been shown to contribute to the disease pathophysiology. In recent years, attention has shifted towards the role that innate lymphoid cells (ILCs) may play in the dysregulation of intestinal immunity observed in IBD. ILCs are a group of heterogenous immune cells which can be found at mucosal barriers. They act as critical mediators of the regulation of intestinal homeostasis and the orchestration of its inflammatory response. Despite helper-like type 1 ILCs (ILC1s) constituting a particularly rare ILC population in the intestine, recent work has suggested that an accumulation of intestinal ILC1s in individuals with IBD may act to exacerbate its pathology. In this review, we summarize existing knowledge on helper-like ILC1 plasticity and their classification in murine and human settings. Moreover, we discuss what is currently understood about the roles that ILC1s may play in the progression of IBD pathogenesis.

## Introduction

Inflammatory bowel disease (IBD) primarily describes two idiopathic conditions characterized by chronic relapsing inflammation in the intestine: ulcerative colitis (UC) and Crohn’s disease (CD). Despite many similarities between the two, UC and CD are differentiated based upon their histopathological features, the primary affected anatomical location, and their associated symptoms. Within the gastrointestinal tract, UC is exclusively colorectal, with mucosal inflammation found continuously extending from the rectum throughout the length of the colon (1). In contrast, individuals with CD exhibit discontinuous pockets of transmural inflammation which can be found scattered along the length of the gastrointestinal tract (2). In comparison to the relatively superficial legions of UC, CD is destructive, with around half of all patients exhibiting complications such as strictures or fistulae, within 10 years of being diagnosed (2). Yet while the precise etiology of IBD remains unknown, its progression has been shown to be influenced by complex interactions between genetic predispositions (3), perturbations to the gut microbiome and its associated metabolites (4–8), and the dysregulation of innate and adaptive immune responses (2, 9). Recently, attention has shifted towards the role that innate lymphoid cells (ILCs) may play in the pathophysiology of IBD. ILCs are a highly heterogenous family of lymphocytes which are considered the innate counterpart of adaptive T cells. Broadly, they have been split into 5 subtypes based on their development, transcription factor expression, and effector functions; these comprise natural killer (NK) cells, lymphoid tissue inducer, and the helper-like type 1, 2 and 3 ILCs (ILC1, ILC2, and ILC3, respectively) (10).

As extensively reviewed elsewhere, the abundance and phenotype of ILCs were shown to vary substantially across tissues (11). However, in contrast to other mucosal sites, such as the salivary gland and liver, ILC1s constitute an extremely rare ILC population in the intestine. Despite this, recent work suggests that they may play a critical role in the pathology of IBD (12–16). However, due to their substantial phenotypic overlap with NK cells, the identification and characterization of intestinal ILC1 remains under debate. Here we will discuss the complexities surrounding the differentiation of ILC1s from NK cells, with a focus on those found in the intestine, and highlight the potential roles of ILC1s in the exacerbation of IBD pathogenesis and its associated complications.

## Murine Intestinal ILC1

In mice, both ILC1s and NK cells can be identified by their shared co-expression of the activating receptors NK1.1 and NKp46 in the absence of other cell lineage (Lin^-^)-defining markers, including CD3, CD14, CD19, and T cell receptor proteins (17) (**Table 1**). Additionally, they share the expression of T-bet, a canonical master transcriptional regulator of type 1 immunity which includes the production of IFN-γ in lymphocytes (19). Within the murine intestine, four distinct populations of Lin^-^ NK1.1^+^ NKp46^+^ cells have been described, which were differentiated based on their anatomical location, transcription factor expression, cell surface marker profile, and effector functions (10). These are comprised of conventional NK (cNK) cells, lamina propria (LP) ILC1s, intra-epithelial ILC1s (ieILC1), and a population of ILC1-like ex-ILC3s.

**Table 1:** NK cell and ILC1 Markers.

Mouse				Human				
	LP ILC1	ieILC1	cNK cells		LP ILC1	ieILC1	cytotoxic ILC1 / trNK cells	cNK cells
**NK1.1**	+	+	+	**CD161**	+	lo	+/lo	–
**NKp46**	+	+	+	**NKp46**	+/-	+	+/lo	+
**T-bet**	+	+	+	**T-bet**	+/-	+/-	+	+
**Eomes**	–	+	+ ^↓^	**Eomes**	–	+	+/-	+
**CD127**	+	–	–	**CD127**	+	–	+/-	–
**CD49a**	+/-	+	- ^↑^	**CD49a**	–	+	+	–
**c-Kit (CD117)**	+	+	–	**c-Kit (CD117)**	–	–	+/-	–
**CD49b (DX5)**	–	–	+ ^↓^	**NKp44**	–	+	+/-	–
**CD90 (Thy1)**	+	lo	- ^↑^	**CD94**	+/-	lo	+/-	+
**KLRG1**	-/lo	–	+	**KLRG1**	+	nd	+	+
**CD103**	–	+	–	**CD103**	–	+	+/-	–
**CXCR6**	+	+	-/lo	**CXCR6**	nd	+	lo/-	–
**Ly49D/H**	-/lo	–	+/-	**KIR**	–	+	+/-	+
**Ly6C**	-/lo	-	+	**NKG2A**	–	+	lo	lo
**CD160**	lo	+	- ^↑^	**CD160**	–	+/-	–	- ^↑^
**CD200r1**	+/-	+/-	–	**CD200r1**	+	lo	+*	–
**Tcf-7**	–	–	-/lo	**CD56**	–	+	+	+
**CD11b**	**-**	-	+	**CD11b**	–	+/lo	lo	+
**HOBIT**	+	+	–	**HOBIT**	+	+	–	–
**TRAIL**	nd	nd	- ^↑^	**IKZF3 (Aiolos)**	+	+	+	+
**CD122**	lo	nd	+	**CD122**	lo	+	+	+
**CD61**	+	nd	- ^↑^	**CD16**	nd	–	+/-	- ^↑^
**CD69**	+	+	- ^↑^	**CD69**	+/-	+	+	-/lo
**Granzyme**	–	+	+	**Granzyme**	–	+	+	+
**Perforin**	–	+	+	**Perforin**	–	+	+	+

Markers which may be used to identify and discriminate between NK cells and ILC1sin mice with a C57BL/6 background, and human tissues. Mouse LP ILC1 and ieILC1 markers shown here are those which have been determined in the intestine and these markers may vary between tissues. For both mouse and human cNK cells and iNK cells, these are primarily circulating cells and so these markers were predominantly identified from cells isolated from the blood. Human cytotoxic ILC1s/trNK cells are exclusive to those identified in the intestine; however, due to the limited information on human intestinal LP ILC1s and ieILC1 populations, these markers additionally include those found in human tonsils, where the majority of research into human ILC1s has been performed, and which have been shown to exhibit substantial overlap with those found in the intestine (12–14). Abbreviations: Lamina propria (LP), intra-epithelial ILC1s (ieILC1), tissue-resident (tr)- , conventional (c)-, and immature (i)-NK cells. Annotations: (+) positive expression; (-) not expressed; (+/-) heterogenous expression; (lo) expressed, but to a low level; (nd) not determined in the specific tissue/s examined; ^↑^ can be regulated following activation; ^↓^ can be downregulated following activation; * only observed in the CD127^+^ ILC1-like population (18).

Predominantly found circulating in the blood or residing in the bone marrow and lymphoid organs, cNK cells are critical for the recognition and elimination of virus-infected and transformed cells, mediated *via* their secretion of cytotoxic effector molecules, such as perforin and granzyme B, and of proinflammatory cytokines, principally that of IFN-γ (10). In contrast, ILC1s are primarily a tissue-resident population of cells (20, 21). Within the LP, ILC1s harbor little to no cytotoxic capacity but exhibit significantly greater IFN-γ secretion than cNK cells, coupled to the production of TNFα upon their activation (12, 22, 23). Moreover, they lack the characteristic expression of Eomes found in cNK cells, instead expressing the IL-7 receptor subunit CD127, a marker of all helper-like ILC lineages (12, 22, 24–26). In the intestine, homeostatic cNK cells and LP ILC1s have further been shown to exhibit a dichotomous expression of cell surface markers including CD49a, CD49b, CXCR6, and CD200r1 and the inhibitory Ly49 family of receptors (21, 27, 28). Consequentially, under steady-state conditions, murine ILC1s and cNK cells form distinct clusters following global analyses of both their chromatin and transcriptional landscapes (21, 27, 29). However, none of those cited above acts alone as a population-defining marker. The advent of single-cell RNA sequencing technologies has highlighted the complex tissue-specific expression patterns of ILC1s and revealed that, even within tissues, ILC1 populations exhibit substantial transcriptional, functional, and developmental heterogeneity (11, 21, 27, 28, 30). Consequentially, many markers previously proposed to define ILC1s, including CD49a, TRAIL, Ly6C, CD103, CD200r1, and CD127 (10, 11), have been found inconsistently expressed within ILC1 populations and/or have been found expressed in immature NK cells and their progenitors (17, 21). This is exemplified by Eomes. While only cNK cells depend on the expression of Eomes for their maturation (31), a lack of Eomes is insufficient as a marker for ILC1s, as it is not expressed on immature NK cells (26). To complicate matters further, activated cNK cells have been shown to downregulate Eomes expression and upregulate ILC1 markers (32–34), resulting in increasingly blurred boundaries between the cell types under inflammatory conditions—for example, NK cells have recently been shown to be able to transition towards an ILC1-like phenotype (Eomes^-^ CD49a^+^ Ly6C^+^) as a consequence of IL-12 s following infection with *Toxoplasma gondii (* 32), or in response to TGF-β signaling in the tumor microenvironment (35), in a manner which may be mediated *via* STAT4 signaling (36). Consequentially, no universal cell surface marker that is able to conclusively distinguish ILC1s from NK cells has been identified.

As with the ability of cNK cells to differentiate to ILC1s, there is substantial and convincing evidence demonstrating a high level of plasticity between the helper-like ILCs in mice (37), particularly among ILC3s and ILC1s (23, 27, 28, 38, 44). In homeostasis, ILC3s form the major ILC population in the intestine, characterized *via* a high expression of the transcription factor RORγt and secretion of the cytokine IL-22 (10). However, in response to an infection or IL-12 signaling, ILC3s can downregulate RORγt while upregulating the expression of T-bet (38–42, 44), of which ILC1s are completely dependent upon for their differentiation (10) and post-developmental maintenance (43). Consequentially, the IL-22-expressing population of intestinal ILC3s is depleted, replaced by an expansion of IFN-γ-producing cells exhibiting a phenotype indistinguishable from ILC1s. Notably, using RORγt fate mapping analysis, an accumulation of these ILC1-like ex-ILC3s (Lin^-^ Eomes^-^ NK1.1^-^ IFNγ^+^ T-bet^+^) was shown to be the principal driver of *Campylobacter jejuni*-induced intestinal pathology (44).

Finally, the remaining Lin^-^ NK1.1^+^ NKp46^+^ population described in mice are CD103^+^ ILC1s, resembling the well-characterized ieILC1s in the human intestine (27).

## Intestinal ILC1 in Humans

Two distinct types of ILC1s have been described in the human intestine (**Table 1**). Intestinal LP Lin^-^ c-Kit^-^ NKp44^-^ NKp46^+^ CD127^+^ CD161^+^ ILC1s exhibit T-bet expression and produce IFNγ in response to inflammation, resemblant of cNK cells (12). However, human LP ILC1s are Eomes^-^ and do not express perforin or granzyme B or other markers typically seen in cNK cells in humans with cNK cells, including the expression of KIR3DL1, CD56, and/or IL1R1 (12, 13). These intestinal LP ILC1s are resemblant of ILC1s identified within other human mucosal tissues, such as the tonsils (45) where the majority of research into human ILCs have been performed. The second ILC1 subtype is a cytotoxic population found within the intra-epithelial lymphocyte compartment, characterized by their high expression of CD103 relative to other local ILC populations (13, 14). Contrasting those found in the LP, human CD103^+^ ILC1s are NKp46^+^ CD56^+^ Eomes^+^ CD127^-^ CD161^low^, thus exhibiting a transcriptional and effector function signature reflecting that of both cNK cells and LP ILC1s (13, 14, 46).

Importantly, while the presence of NK1.1^+^ NKp46^+^ CD127^+^ ILC1s within the intestinal LP is consistently observed in mice, a question remains as to the existence of their NKp46^+^ CD127^+^ CD161^+^ LP ILC1 counterparts within the non-inflamed human intestine (30, 47, 48). Critically, the level of T-bet expression is heterogeneous in both ILC1 and NK cell populations in human tissues (46, 49), and a lack of T-bet in a putative ILC1 population (Lin^-^ c-Kit^-^ NKp44^-^ CD45^+^ CD127^+^) has led some to question the very existence of ILC1s themselves (47). In contrast, the presence of cytotoxic populations of ILC1s, comparable to ieILC1s (CD103^+/-^ T-bet^+^ Eomes^+^ CD56^+^ NKp46^+^ CD49a^+^ CD127^-^), is more consistently observed in various human tissues (13, 14, 47, 50, 51). However, these constitute highly heterogenous populations of cells which have been proposed to resemble cNK cells more closely than other helper-like ILC lineages (46, 47, 49). Accordingly, they are often instead classed as tissue-resident NK (trNK) cells.

While the subpopulations of LP ILC1, ieILC1, cytotoxic/trNK cells, and cNK cells exhibit highly overlapping phenotypes, a direct transition between these populations in the intestine has yet to be demonstrated. However, as described in mice, ILC3 to ILC1 phenotypic plasticity may also be present in human intestinal tissues (13, 15, 47, 52, 53)—for example, CD14^+^ dendritic cells from CD resection specimens have been found to promote the differentiation of ILC3s into ILC1s *in vitro (* 13). Moreover, human tonsillar ILC3s have shown that they are able to convert to ILC1-like ex-ILC3s in a manner which may be driven, in part, by TGF-β and IL-23 acting coordinately to regulate the expression of the transcription factors Aiolos, T-bet, and RORγt (15, 53). These transitionary cells were also identified in the ileum of both individuals with CD and non-disease controls (15), suggesting that it is not a tissue-specific phenomenon. In the absence of fate mapping within human tissues, this plasticity acts to further complicate the identification of *bona fide* human ILC1s.

The consequence of their overlapping transcriptional phenotypes and the plasticity between populations result in a lack of consistently used nomenclature to define and differentiate between ILC1 and NK cells, particularly within human intestinal tissues. Until markers which conclusively discriminate between the two are identified, this remains a major challenge to the field.

## ILC1 in Intestinal Inflammation

**Figure 1:**
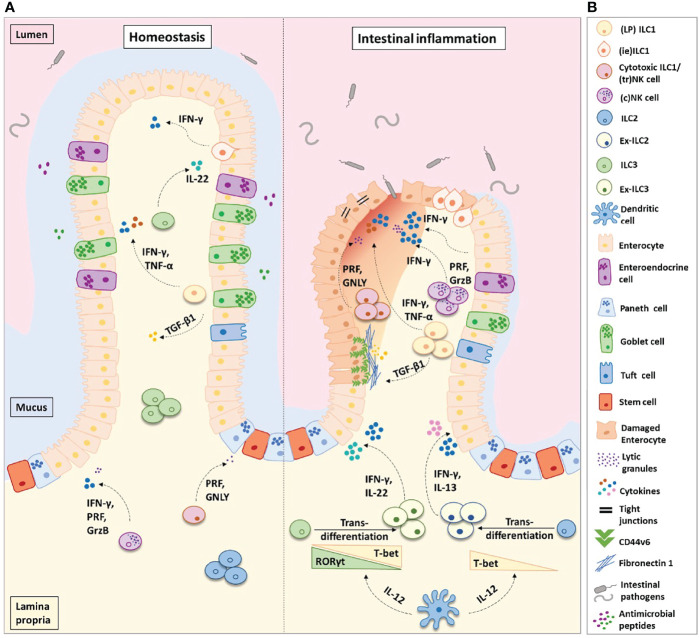
Helper-like type 1 ILCs (ILC1s) in homeostasis and intestinal inflammation. **(A)** ILC1s comprise of lamina propria (LP) ILC1s, intra-epithelial (ie) ILC1s, and cytotoxic ILC1s/tissue-resident (tr) natural killer cells. In the healthy intestinal microenvironment, ILC1s are the least frequent ILC population. In inflammatory bowel disease (IBD), the frequency of ILC1s is increased at the expense of ILC3s and ILC2s. ILC3s and ILC2s transdifferentiate into ILC1s aided by the secretion of IL-12 from dendritic cells. Ex-ILC3s can secrete both IFN-γ and IL-22 upon stimulation, while ex-ILC2s can produce both IFN-γ and IL-13. The increase in ILC1 frequency leads to an increase in pro-inflammatory cytokine secretion and augmented levels of ILC1-derived TGF-β1. TGF-β1 has been shown to increase CD44v6^+^ expression in epithelial and mesenchymal cells. In homeostasis, this may aid wound healing; however, in IBD this is associated with enhanced fibronectin-1 deposition and extracellular matrix degradation, consequently promoting tissue scarring. Abundant levels of IFN-γ disrupt the epithelial tight junctions, leading to increased epithelial permeability. This allows commensal, pathogenic, and opportunistic bacterial species (such as *Bacteroides fragilis*, *Escherichia coli*, *Mycobacterium avium* subsp. *paratuberculosis*, and *C. difficile*) to penetrate the lamina propria, thus perpetuating the inflammation. **(B)** Figure key illustrating the different cell types and molecules participating in intestinal inflammation. IFN-γ, interferon-gamma; IL-22, interleukin-22; IL-17, interleukin-17; IL-13, interleukin-13; IL-12, interleukin-12; TGF-β1, transforming growth factor-beta 1; TNF-α, tumor necrosis factor-alpha; PRF, perforin; GrzB, granzyme-B; GNLY, granulysin.

LP ILC1s act in the first line of defense to external pathogens, rapidly inducing cytokine secretion upon their activation and promoting the recruitment of other immune cells to the site of inflammation (10) (**Figure 1**). Most studies addressing the potential roles of LP ILC1s in murine intestinal inflammation are primarily focused on their secretion of pro-inflammatory cytokines, namely, IFN-γ and TNFα (14, 18, 33, 38, 39, 44, 54). While these have been shown to aid the clearance of infections, such as mouse cytomegalovirus (33), *Toxoplasma gondii (* 23), and *Clostridium difficile (* 54), the excess production of IFN-γ and TNFα is highly disruptive to intestinal homeostasis, inhibiting proliferation and inducing apoptosis of local epithelial cells (55, 56). Consequentially, signaling *via* these pro-inflammatory cytokines has been shown to exacerbate IBD-associated pathology in response to infection with *Helicobacter typhlonius* and in mouse models of dextran sulfate sodium (DSS)-induced colitis (12, 25, 43, 55, 57–59)—for example, in response to DSS treatment, IFN-γ disrupts the vascular barrier integrity by altering the adherens junction protein VE-cadherin. As a result, this drives an increase in vascular permeability and an accumulation of immune cells at the site of inflammation, enabling the migration of intestinal bacteria into the blood and other organs (55). Concordantly, the depletion of LP ILC1s and/or their associated cytokines have been shown to significantly attenuate the symptoms of colitis *in vivo (* 43, 57, 59).

As observed in mice, VE-cadherin-mediated disruption of the intestinal vascular barrier by IFN-γ has additionally been described in human IBD patients, suggesting a direct causative role of IFN-γ in the pathogenicity of the disease (55). Moreover, an accumulation of ILC1s in the inflamed intestine has repeatedly been identified in patients with IBD (12–14, 18, 52, 60). This was first observed by Bernink *et al.*, who demonstrated an accumulation of *IFNG*-expressing c-Kit^-^ NKp44^-^ CD127^+^ ILC1s in the intestinal LP of Crohn’s disease patients and following DSS-induced colitis in mice with a humanized immune system (12). Subsequently, these results have been confirmed in larger patient groups (15, 60) and have been shown not to be restricted to LP ILC1s but to include an increase in the proportions of cytotoxic ieILC1s/trNK cells and ILC1-like cells (CD127^+^ CD94^+^) in individuals with CD (14, 18). However, it is not currently clear whether an increase in the frequencies of ILC1s is a universal feature of IBD. Recent investigations into ILC1s in the mucosal tissue of patients newly diagnosed with CD and UC found that while LP ILC1 proportions increased at the sites of active inflammation in CD, ILC2s instead accumulated in patients with UC (48). This finding supports the controversial Th1/Th2 paradigm, in which CD is a Th1-mediated disease and UC a Th2-mediated disease (61). In contrast, using cytometry by time-of-flight analysis, the presence of NK cells and ILC1s (Lin^-^ CD161^+^ T-bet^+^ CD56^lo-^) has been demonstrated in the mucosa of UC patients, albeit to a lesser extent than was present in individuals with CD (62).

In patients with active IBD, perturbations in the ILC compartment may not be restricted to the intestine, with a small increase in the frequency of c-Kit^-^ NKp44^-^ CD127^+^ CD161^+^ ILC1s identified in their peripheral blood (63). However, this finding is not universal with earlier reports demonstrating no alterations in circulating ILC subtypes between IBD patients and/or healthy controls (48, 62). Recently, in the absence of changes in the total frequencies of helper-like ILCs, an expansion of those exhibiting putative markers of activation was observed, including HLR-DR^+^ ILC1s and both HLR-DR^+^ and FMALF1^+^ ILC2s (64). This suggested that existing peripheral ILC populations may be switching from a homeostatic to an activated phenotype. While a question remains as to what extent this may impact IBD pathogenesis (65), it is notable that such a switch was additionally observed in the population of ILC precursors (ILCp); an increase in NKp44^+^ and CD56^+^ ILCp was detected in CD patients, coupled to a decrease in their corresponding naïve CD45RA^+^ ILCp (64). However, while circulating human ILCp has been shown to contribute to the pool of ILCs in tissues (66), the extent to which perturbations in peripheral ILCp in IBD patents may impact the ILC compartment within the intestinal mucosae remains to be determined—for example, recent research has shown that CD45RA^+^ ILCp exhibits tissue-specific differentiation potential (67). Moreover, the authors demonstrated an accumulation of a CD62L^-^ subpopulation of CD45RA^+^ ILCp in the inflamed mucosa of patients with both UC and CD. The expansion of CD26L^-^ ILCp was found to be inversely proportional to the frequency of NKp44^+^ ILC3s, suggesting that disruptions to ILCp compartments may act to promote the disequilibrium of ILCs observed in IBD (67).

## A Role for ILC1-Derived TGF-β in IBD Pathogenesis?

The lack of long-term treatment options for IBD brings into question the principal role of pro-inflammatory cytokines as key drivers of the disease pathology—for example, the current gold-standard treatment is the use of TNF-α blockers (*e*.*g*., infliximab, adalimumab, certolizumab pegol, and golimumab), yet while this has dramatically improved the IBD outcomes for both CD and UC patients (68), only 1 in 3 initially responded to the drug, and in 30–50% of these patients, the efficacy of treatment decreases over time (69). Furthermore, despite IFN-γ signaling being shown to drive colitis in mice, a lack of efficacy was observed in treatments aimed at suppressing signaling by this pro-inflammatory cytokine in humans (70, 71). However, recent work within our laboratory has demonstrated an alternative signaling method by which ILC1s may act to exacerbate IBD-associated pathology. Using both human and murine intestinal organoids co-cultured with ILC1s, it was shown that LP ILC1-derived TGF-β can drive the degradation of the extracellular matrix, enhance fibronectin deposition, and induce the non-specific expansion of intestinal epithelial cells (16). While in homeostasis these pathways may drive epithelial regeneration and wound healing, under conditions of chronic inflammation, they have the potential to be pathological (15)—for example, TGF-β has previously been characterized as an upstream regulator of fibrosis in CD (72, 73). Notably, despite the organoids used here being genetically, epigenetically, and environmentally identical, pathological matrix remodeling was only observed in the hyper-proliferative ILC1s isolated from the inflamed tissue of CD patients (16). This concurred with previous research in 2019 by Cella et al., who observed a significant increase in a population of proliferative ILC1s in the ileum of individuals with CD (15). These data together highlight a novel non-canonical function of ILC1 in the exacerbation of IBD, in which their augmented expansion may be coupled to enhanced TGF-β signaling, consequentially driving the inflamed tissues toward fibrotic scar tissue formation and/or promoting the growth of epithelial tumors during chronic inflammation, two life-threatening sequalae of IBD (74).

Interestingly, Cella et al. additionally identified a transitionary population of ILC3–ILC1 cells in both CD patients and non-disease controls. As with the conversion of NK cells to ILC1s in the TGF-β-rich tumor microenvironment (35), this transition was demonstrated to be mediated *via* TGF-β signaling in both mice and humans (15, 42). Considering that the accumulation of ILC1s in IBD is coupled to a decrease in the frequency of ILC3s (12, 48, 60), a question remains as to what extent this is reflective of an expansion of the existing ILC1 population or an increased conversion of other ILC subtypes to an “ILC1-like” phenotype or both. Moreover, TGF-β has also been shown to impair NK cell activity (75), and the presence of dysregulated NK cells has been observed in the blood of IBD patients (76). Thus, while these data do not show a direct relationship to intestinal inflammation, they highlight the need for further investigations into the pleiotropic role of TGF-β signaling in the dysregulated immune compartment found within IBD.

## Concluding Remarks and Future Directions of Research

ILC1s represent a minor fraction of the innate cell population in the intestinal LP of healthy individuals. When present in small numbers, ILC1-derived TGF-β may contribute to the maintenance of epithelial regeneration (16). However, the expansion of their population at sites of active intestinal inflammation may indicate their potential role as inducer and/or driver of IBD pathogenicity, which is linked to the development of IBD sequelae such as fibrosis and cancer (16). Critically, there are still many key questions that need to be addressed regarding their presence in inflamed tissues. Do ILC1s accumulate in the intestinal lamina propria of CD patients as a cause or consequence of inflammation? Does the pro-inflammatory microenvironment lead to the augmented plasticity of ILC2s and ILC3s, driving their transition towards ILC1s? Or does it promote ILC1 survival and expansion *via* the secretion of the cytokines and growth factors that they depend on? To what extent may perturbed blood ILC compartments impact or reflect on those observed within the intestine? Does the inflamed microenvironment observed in CD imprint a highly proliferative state on ILC1s? Are the effects on ILC1s a result of “trained” immunity as recently reported for murine ILC3 (77)?

The development of new experimental techniques, such as the co-culture of human organoids with ILCs (78, 79) and single-cell multiomics, provides new avenues to address these questions. Such studies will lead to a better understanding of the role of ILC1 in IBD. Importantly, they will contribute to the development of new therapeutic approaches to revert the pathogenic accumulation of ILC1s in inflamed tissues.

## Author Contributions

JN and DC conceptualized the manuscript’s theme. DC, IC, LR, and JN designed and reviewed the manuscript. DC and IC researched and wrote the manuscript. DC designed the figure. IC constructed the table. All authors contributed to the article and approved the submitted version.

## Conflict of Interest

The authors declare that the research was conducted in the absence of any commercial or financial relationships that could be construed as a potential conflict of interest.

## Publisher’s Note

All claims expressed in this article are solely those of the authors and do not necessarily represent those of their affiliated organizations, or those of the publisher, the editors and the reviewers. Any product that may be evaluated in this article, or claim that may be made by its manufacturer, is not guaranteed or endorsed by the publisher.
